# Efficacy and Safety of Calcium Hydroxylapatite for Nasal Augmentation: A 12‐Month Prospective Study in a Chinese Population

**DOI:** 10.1111/jocd.70862

**Published:** 2026-04-21

**Authors:** Ji Wang, Wuhan Wei, Junsheng Chen, Aijun Zhang, Peisheng Jin, Yanping Guo

**Affiliations:** ^1^ Xuzhou Central Hospital Xuzhou Jiangsu People's Republic of China; ^2^ Center of Plastic and Aesthetic Surgery, Affiliated Hospital of Xuzhou Medical University Xuzhou Jiangsu People's Republic of China; ^3^ Plastic Surgery Hospital, Chinese Academy of Medical Sciences, Peking Union Medical College Beijing People's Republic of China

**Keywords:** biostimulatory material, calcium hydroxylapatite, mid‐face rejuvenation, nasal augmentation

## Abstract

**Background:**

Calcium hydroxylapatite (CaHA) is a biostimulatory material that induces collagen neogenesis, but its long‐term volumetric dynamics following nasal injection remain insufficiently characterized. This study aimed to evaluate the efficacy and safety of CaHA gel for nasal augmentation in a Chinese population.

**Methods:**

In this prospective study, 24 female patients received CaHA injections for nasal augmentation. Efficacy was assessed with three‐dimensional (3D) facial scanning at 0 (immediately after injection), 1, 3, 6, 9, and 12 months. Patient satisfaction was evaluated using the Global Aesthetic Improvement Scale (GAIS). Safety was assessed by the Numerical Rating Scale (NRS) for injection‐related pain and recording of injection site adverse reactions.

**Results:**

All 24 patients completed the 12‐month follow‐up. 3D volumetric analysis revealed a transient reduction from Month 0 to Month 1 (*p* < 0.05), followed by a modest numerical increase from Month 1 to Month 6 that did not reach statistical significance (*p* > 0.05). At Month 12, the volume was 0.94 ± 0.45 mL, representing a significant decline compared with Months 6 and 9 (*p* < 0.05). The GAIS responder rate (subjects rating “Much improved” or “Very much improved”) was 79% at Month 3 and 58% at Month 9. Injection‐related pain was mild (mean NRS at 0 min: 1.29) and resolved within 60 min. Adverse events were transient, primarily swelling (60.5%) and tenderness (21.1%), resolving spontaneously within 1 week. No serious complications occurred.

**Conclusions:**

CaHA injection is an effective and safe treatment for nasal augmentation, providing durable volumetric improvement and high patient satisfaction over 12 months.

## 1 Introduction

Facial rejuvenation and contour optimization have become increasingly prominent in aesthetic plastic surgery, with nasal augmentation standing out as one of the most popular procedures to enhance facial harmony and aesthetic proportion [1, 2]. The injectable fillers have revolutionized nonsurgical rhinoplasty, offering a minimally invasive alternative to traditional surgical techniques, with advantages of shorter recovery time, adjustable outcomes, and reduced intraoperative trauma [3, 4]. Among various injectable materials, hyaluronic acid (HA) fillers have various advantages, such as excellent biocompatibility, predictable molding property, and reversibility via hyaluronidase [4, 5]. However, the inherent limitations of HA, including linear enzymatic degradation, insufficient rigidity and viscosity for long‐term structural support in the nasal dorsum, and the need for repeated injections to maintain results, have prompted the exploration of alternative biostimulatory fillers [6]. These limitations are particularly relevant in the nasal region, an anatomically complex area with unique biomechanical properties where fillers must provide structural support against the pressure of skin and soft tissues on the injected area [7, 8].

Calcium hydroxylapatite (CaHA) has emerged as a promising biostimulatory material in facial aesthetic procedures [9]. Composed of synthetic CaHA microspheres suspended in a biocompatible carrier gel (typically sodium carboxymethyl cellulose, CMC, with lidocaine for analgesia), this material acts as a biodegradable scaffold that recruits macrophages and activates dermal fibroblasts, thereby stimulating the synthesis of collagen [9, 10]. Several studies demonstrate that over time, the carrier gel is absorbed and the CaHA microspheres become encapsulated by newly formed connective tissue, with collagen fibers providing a durable and natural‐looking augmentation [9, 10, 11]. Unlike HA fillers that rely solely on the physical presence of the material, CaHA‐induced neocollagenesis enables long‐term volume maintenance and natural tissue integration, making it suitable for facial regions requiring structural support, such as the nasolabial folds, jawline, and cheekbones [10, 12, 13]. A pivotal multicenter, randomized controlled trial, alongside subsequent meta‐analyses, confirmed CaHA's superior longevity in nasolabial folds compared to several HA fillers, with meaningful volume retention and higher patient satisfaction observed at and beyond 12 months [12].

Despite the proven efficacy of CaHA in other facial subunits, its application in nonsurgical nasal augmentation remains insufficiently characterized. The nose is an anatomically complex region with distinct biomechanical properties, requiring fillers to balance immediate contouring effect, long‐term structural stability, and safety in a high‐risk vascular zone [5, 6]. Existing studies on CaHA for nasal augmentation lack objective quantification of volumetric change. The dynamic volumetric changes of CaHA in the nasal region have not been systematically validated with high‐resolution three‐dimensional (3D) scanning. This technology offers a more precise and objective assessment than traditional clinical photography, as it enables quantitative volume measurement and digital superimposition of pre‐ and posttreatment models, a method proven highly accurate in other craniofacial applications [8, 14, 15, 16].

Therefore, the present study aimed to evaluate the efficacy, safety, and volumetric dynamics of CaHA for nasal augmentation over a 12‐month follow‐up period. The findings of this study are expected to provide clinical evidence for the application of CaHA in nonsurgical rhinoplasty, expand the understanding of its volumetric dynamics in the anatomically complex nasal region, and validate its versatility beyond previously studied facial subunits.

## 2 Methods

### 2.1 Study Design and Treatment Procedure

**FIGURE 1:**
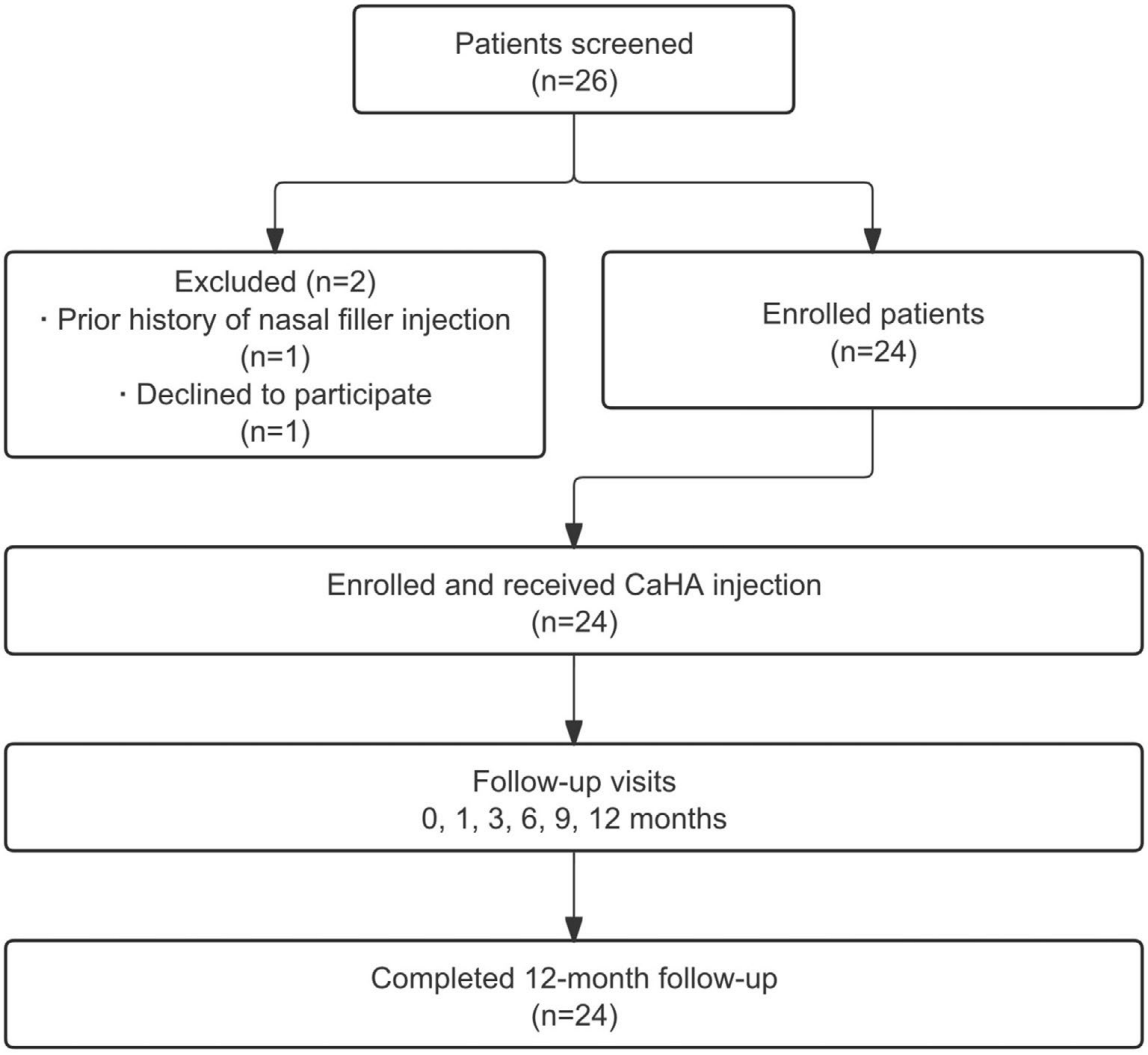
Patient flow diagram. A total of 26 patients were screened for eligibility. Two patients were excluded (one had a prior history of nasal filler injection, and one declined to participate), and the remaining 24 patients underwent nasal augmentation with CaHA and completed all scheduled 12‐month follow‐up visits.

From April 2024 to June 2024, a total of 26 patients seeking nasal augmentation were screened. Of these, two patients were excluded; one had a prior history of nasal filler injection, and one declined to participate. The remaining 24 patients were enrolled and underwent nasal augmentation via injection of CaHA gels (Figure 1). All patients provided written informed consent, and the protocol was approved by the Ethics Committee of the Affiliated Hospital of Xuzhou Medical University (approval number: XYFY2024‐QL100).

The injected gel used was a calcium hydroxylapatite (CaHA) gel (Beierkang Biomedical Technology Co. Ltd., Shanghai, China). It consists of 30% synthetic CaHA microspheres (25–45 μm in diameter) suspended in a carrier gel of sodium carboxymethyl cellulose (CMC), glycerin, and sterile water, premixed with 0.3% lidocaine.

**FIGURE 2:**
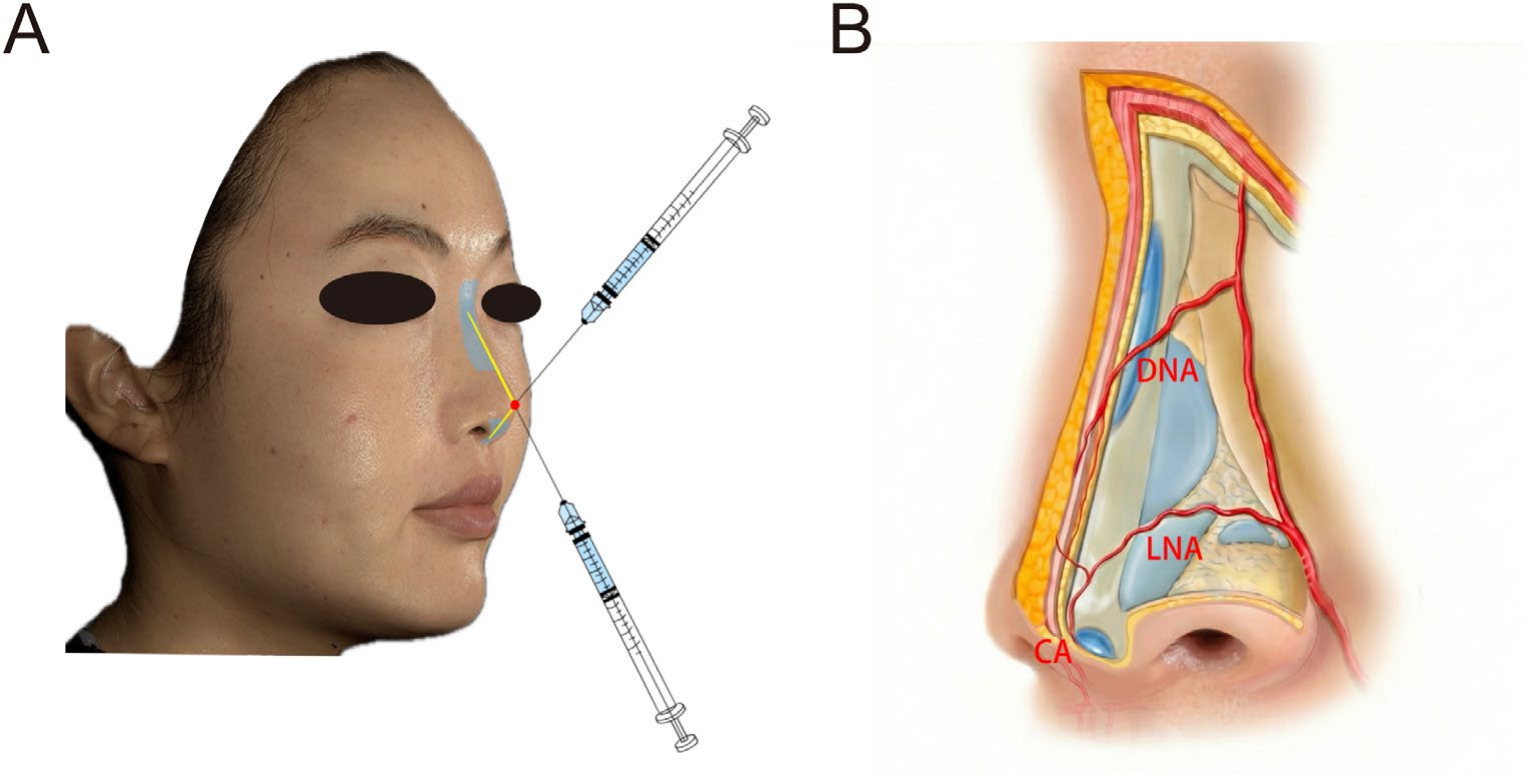
Schematic diagram of CaHA injection in nasal augmentation. (A) Schematic representation of nasal augmentation using a blunt cannula at the supraperiosteal plane. The red dot indicates the injection entry point at the nasal tip. The yellow line indicates the midline placement of the CaHA product. (B) High‐risk arterial zones in the subcutaneous layer: Lateral nasal artery (LNA), dorsal nasal artery (DNA), and columellar artery (CA). CaHA (blue) is deposited in the sub‐SMAS plane along the dorsum and columella, with careful avoidance of these arteries.

All injections were performed by experienced physicians following a standardized protocol. CaHA gel was administered via periosteal or supraperiosteal approach to three nasal subunits: the nasal dorsum, columella, and anterior nasal spine (Figure 2). The nasal midline and the entry point at the nasal tip were marked prior to the procedure. Local infiltration anesthesia was administered at the marked entry point. Injections were delivered using either a 23‐G cannula, a 50 mm cannula for the nasal dorsum, and a 38 mm cannula for the columella and anterior nasal spine. Nasal dorsum: Using a linear retrograde threading technique, CaHA was deposited slowly along the midline in close contact with the underlying periosteum or cartilage, not exceeding 0.1 mL/cm. Columella: From the same entry point, the cannula was directed vertically toward the columellar base. A total of 0.1–0.2 mL CaHA was injected in a retrograde manner along the supraperichondrial plane. Anterior nasal spine: Following columellar injection, the cannula was withdrawn to the anterior nasal spine, where an additional approximately 0.1 mL CaHA was deposited in the supraperiosteal plane to enhance tip projection. The total CaHA injection volume per session did not exceed 2.0 mL. Immediately after injection, gentle manual molding was performed to contour the material and achieve the desired aesthetic outcome. Follow‐up visits were scheduled at months 0 (immediately after injection), 1, 3, 6, 9, and 12 after the treatment. Three‐dimensional (3D) facial scanning and standardized clinical photography were performed before treatment and at each subsequent follow‐up.

### 2.2 Efficacy Assessment

Nasal volume was objectively quantified with 3D facial scanning. A handheld structured‐light three‐dimensional facial scanning system was used to acquire high‐resolution facial surface data. Volumetric analysis was performed using dedicated image‐processing software provided with the system. To calculate the volume change at each follow‐up, the 3D model from that visit was digitally superimposed onto and compared against the post‐procedure baseline model, yielding precise volumetric difference measurements specifically for the nasal region.

Patient‐assessed aesthetic improvement was evaluated using a 5‐point Global Aesthetic Improvement Scale (GAIS). According to the scale, 1 represents “very much improved”, 2 represents “much improved,” 3 represents “improved,” 4 represents “no change,” and 5 represents “worse.” Patients who self‐rated as “much improved” and “very much improved” were classified as treatment responders.

### 2.3 Injection‐Related Pain Assessment

Pain associated with the injection procedure was prospectively assessed using the Numeric Rating Scale (NRS). The NRS is an 11‐point scale ranging from 0 to 10, where 0 represents “no pain”, 1–3 represents “mild pain”, 4–6 represents “moderate pain”, and 7–10 represents “severe pain.” To capture the immediate and short‐term pain profile, assessments were conducted at three predefined time points: immediately after the injection procedure (0 min), and at 30 min and 60 min postinjection. All pain assessments were performed by a dedicated research coordinator in a standardized, quiet setting to minimize bias.

### 2.4 Safety Assessment

Patients filled in a 14‐day safety diary card after each treatment to record the duration and severity of injection site adverse reactions, including pain, redness, tenderness, swelling, bruising, itching, and hard lumps.

### 2.5 Statistics

Data are presented as mean ± standard deviation for continuous variables and as frequencies (percentages) for categorical variables. Continuous variables were analyzed using the repeated‐measures analysis of variance (ANOVA). All analyses were performed with Prism software (version 10.0), and a two‐sided *p* < 0.05 was considered statistically significant.

## 3 Results

### 3.1 Demographic and Treatment Characteristics

**FIGURE 3:**
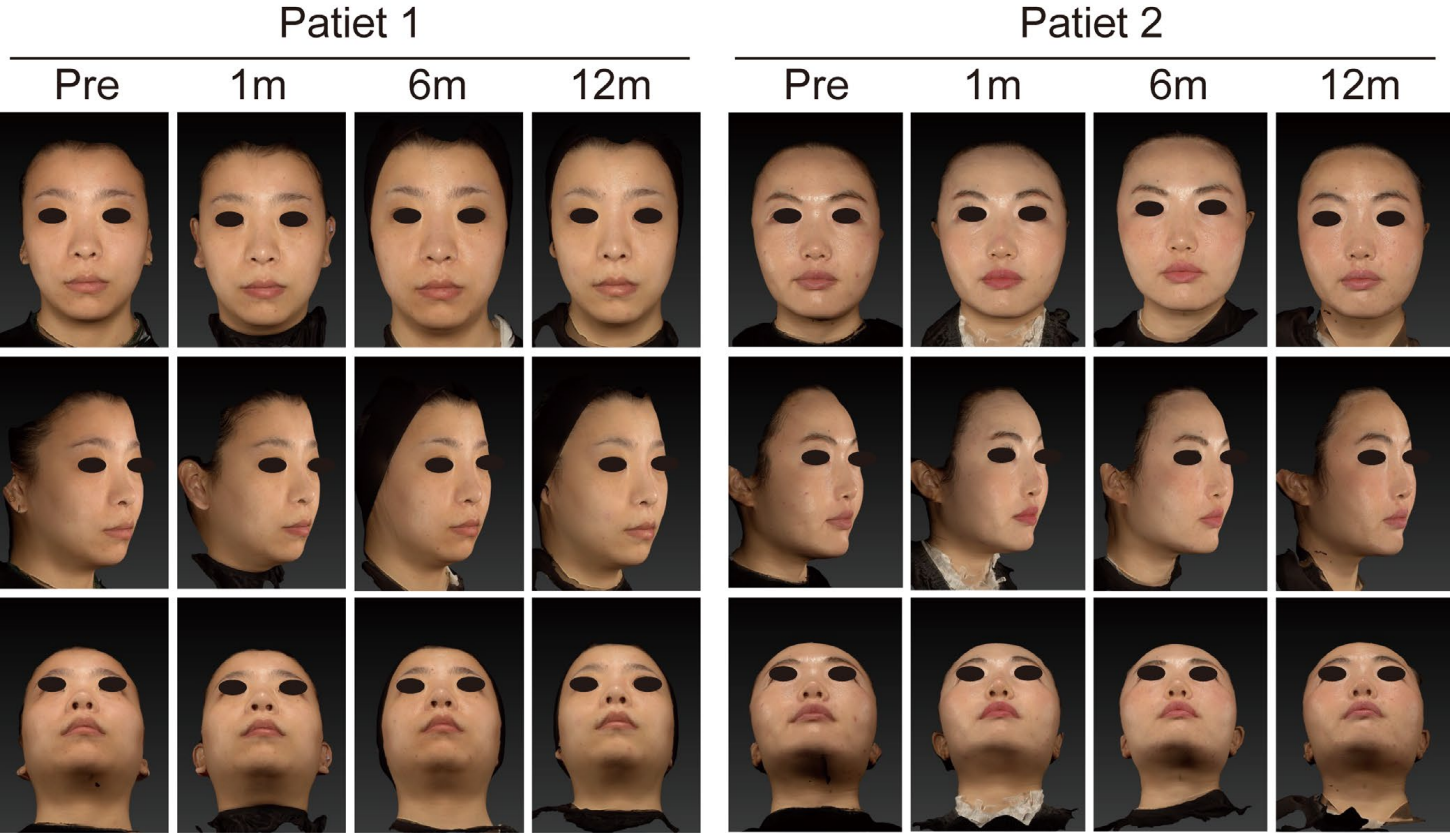
Representative clinical photographs of two patients who received nasal augmentation with CaHA. Images were obtained at baseline (pretreatment), and at Months 1, 6, and 12 following the procedure. For each time point, frontal, lateral (45‐degree), and basal views are presented to comprehensively illustrate the nasal contour changes.

All 24 enrolled patients in the experimental group completed the 12‐month study with no loss to follow‐up (Figure 1). No study visits were missed, and no patients withdrew from the study. Demographic and treatment characteristics are summarized in Table 1. The study population was female patients with a mean age of 37.17 ± 11.42 years (ranging from 22 to 62 years). The mean total injection volume of CaHA gel was 0.93 ± 0.22 mL, with more than half (54.2%) receiving a dose between 0.9 and 1.0 mL. Figure 3 shows the representative photographs of patients at each follow‐up time period.

**TABLE 1:** Demographic and treatment data of patients.

Characteristic	Value
Age (years)	
Mean ± SD	37.17 ± 11.42
Range	22–62
Gender, *n* (%)	
Female	24 (100)
Total injection volume (mL)	
Mean ± SD	0.93 ± 0.22
Range	0.5–1.4
Injection volume category, *n* (%)	
≤ 0.8 mL	7 (29.2)
0.9–1.0 mL	13 (54.2)
≥ 1.1 mL	4 (16.6)

### 3.2 Nasal Volumetric Change

**FIGURE 4:**
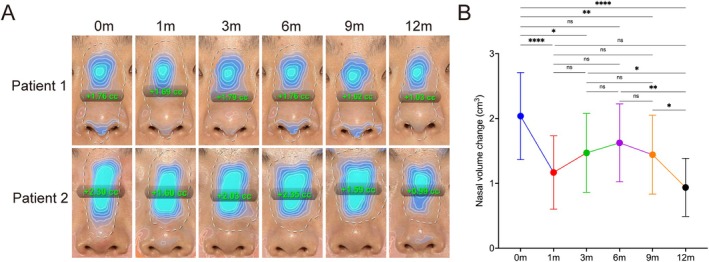
Three‐dimensional volumetric analysis of nasal changes following CaHA augmentation. (A) Representative 3D reconstructions of two patients at baseline and each follow‐up time point (Months 0, 1, 3, 6, 9, and 12 posttreatment). Color mapping (blue) indicates regions of volume increase relative to baseline, visualizing the dynamic volumetric changes over time. (B) Quantitative analysis of nasal volume changes over the 12‐month follow‐up period. *p < 0.05; **p < 0.01; ****p < 0.0001; ns, *p* > 0.05 versus baseline.

Three‐dimensional volumetric analysis revealed a dynamic evolution of nasal contour following CaHA gel injection (Table 2, Figure 4A and Figure S1). At Month 1, the volume was significantly lower than the immediate post‐injection value (2.04 ± 0.67 mL vs. 1.17 ± 0.57 mL, *p* < 0.05). From Month 1 through Month 6, volume showed a modest numerical increase, from 1.17 ± 0.57 mL at Month 1 to 1.62 ± 0.60 mL at month 6. However, this change did not reach statistical significance (*p* > 0.05 for all pairwise comparisons among Months 1, 3, and 6, Table S1, Figure 4B). Notably, the individual patient trajectories revealed that 67% (16/24) of patients had a higher volume at Month 6 than at Month 3, and 75% (18/24) had a higher volume at Month 6 than at Month 1 (Figure S1). Additionally, the volume at Month 6 was not significantly different from the immediate postinjection volume (*p* = 0.15), reflecting the regenerative compensation of CaHA. After Month 6, volume gradually declined. Although no significant difference was observed between Month 6 and Month 9 (*p* = 0.89), a significant decrease was detected by Month 12 compared with Month 6 (*p* < 0.01) and Month 9 (*p* = 0.04) (Table S1, Figure 4B).

**TABLE 2:** Nasal volume changes after CaHA gel injection.

Time point after last injection	Mean volume change ± SD (mL)	Median (mL)	Range (mL)
Immediate (0 m)	2.04 ± 0.67	2.14	0.97–3.33
1 Month (1 m)	1.17 ± 0.57	1.09	0.30–2.76
3 Months (3 m)	1.47 ± 0.61	1.36	0.32–3.05
6 Months (6 m)	1.62 ± 0.60	1.64	0.25–2.94
9 Months (9 m)	1.44 ± 0.61	1.42	0.53–2.84
12 Months (12 m)	0.94 ± 0.45	0.83	0.25–2.00

### 3.3 Patient‐Assessed Global Aesthetic Improvement Scale (GAIS)

**FIGURE 5:**
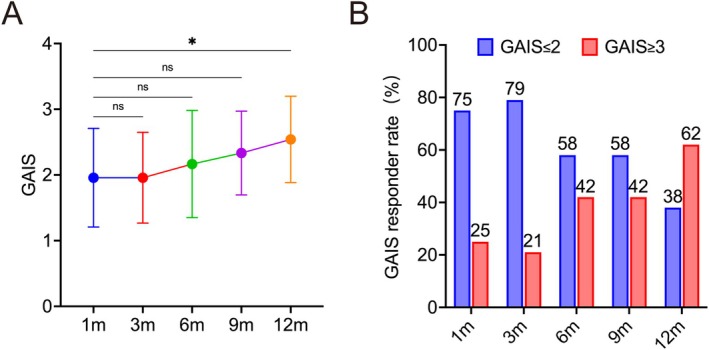
Patient‐reported outcomes assessed by the Global Aesthetic Improvement Scale (GAIS). (A) Patients assessed GAIS scores at Months 1, 3, 6, 9, and 12 following CaHA injection. *p < 0.05; ns, *p* > 0.05 versus Month 1. (B) GAIS responder rates over the 12‐month follow‐up period. Patients were categorized as responders (GAIS ≤ 2, indicating “much improved” or “very much improved”) and nonresponders (GAIS ≥ 3, indicating “improved,” “no change,” or “worse”).

In terms of the patient‐assessed GAIS, the mean GAIS score demonstrated a gradual increase over the 12‐month follow‐up period, from 1.96 ± 0.75 at 1 month to 2.54 ± 0.66 at 12 months (Table 3, Figure 5A), indicating a progressive decline in satisfaction among patients following CaHA nasal augmentation treatment over time. The responder rate remained consistently high throughout all observation time points. The proportion of subjects rated as “much improved” or “very much improved” (GAIS ≤ 2) was highest at 3 months (79%) and remained substantial at 6 months and 9 months (58%) (Figure 5B).

**TABLE 3:** Patient‐assessed Global Aesthetic Improvement Scale over time.

Time point	GAIS, mean ± SD	*p*
1 Month (1 m)	1.96 ± 0.75	
3 Months (3 m)	1.96 ± 0.69	> 0.99
6 Months (6 m)	2.17 ± 0.82	0.78
9 Months (9 m)	2.33 ± 0.64	0.26
12 Months (12 m)	2.54 ± 0.66	0.02

### 3.4 Numeric Rating Scale (NRS)

**FIGURE 6:**
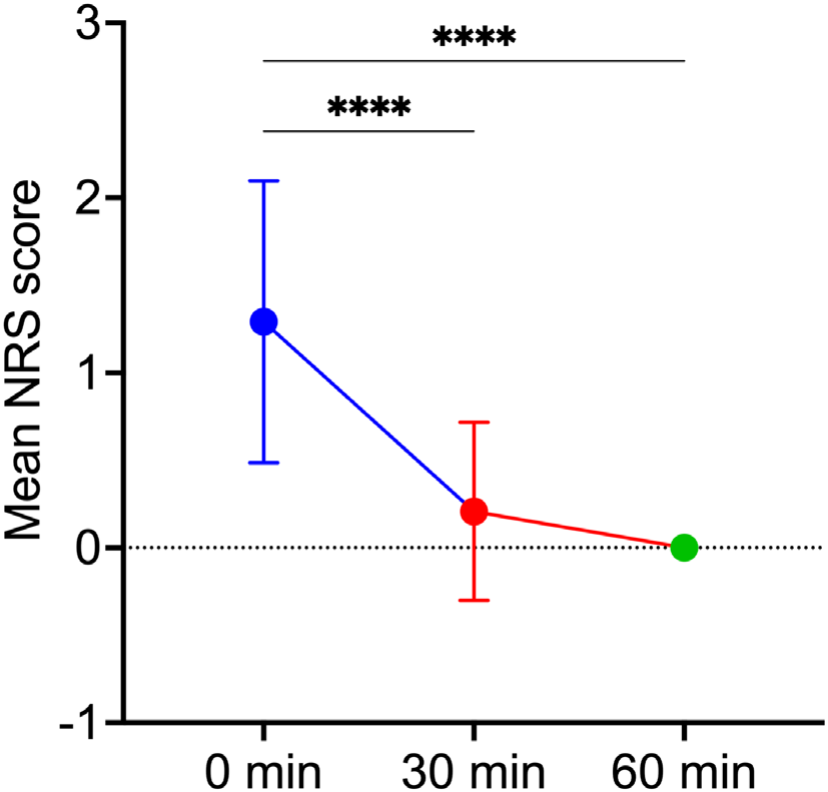
Injection pain assessment using the Numeric Rating Scale (NRS). NRS scores were recorded immediately postinjection (0 min), and at 30 and 60 min following the CaHA injection. ****p < 0.0001 vs. 0 min.

Regarding pain caused by the injection procedure, the mean Numeric Rating Scale (NRS) score immediately after CaHA injection (0 min) was 1.29 ± 0.81, which falls within the range of mild pain. The discomfort subsided rapidly and significantly thereafter. At 30 min post‐procedure, the mean score decreased to 0.21 ± 0.51 (*p* < 0.0001, vs. 0 min), indicating that pain had virtually resolved. By the 60‐min assessment, the mean NRS score had declined to zero (*p* < 0.0001, vs. 0 min), demonstrating complete resolution of injection‐related pain within 1 h (Figure 6).

### 3.5 Safety Evaluation

Local injection site reactions following CaHA nasal augmentation treatment are summarized in Table 4. Swelling was the most frequently reported reaction, occurring in 23 patients (60.5%), with a mean duration of 4.4 ± 1.8 days. Tenderness was reported by 8 patients (21.1%), lasting an average of 5.5 ± 1.8 days. Other transient reactions included redness (7.9%, mean duration: 4.7 ± 3.1 days), pain (5.3%, mean duration: 4.5 ± 2.1 days), and bruising (5.3%, mean duration: 3.5 ± 0.7 days). No cases of itching or hard lumps were observed during the study period. All reported reactions were mild in severity, self‐limiting, and resolved spontaneously within 3 to 8 days.

**TABLE 4:** Local injection site reactions.

Adverse reactions	Incidence, *n* (%)	Mean duration ± SD (Days)	Range (Days)
Pain	2 (5.3)	4.5 ± 2.1	3–6
Redness	3 (7.9)	4.7 ± 3.1	2–8
Tenderness	8 (21.1)	5.5 ± 1.8	3–8
Swelling	23 (60.5)	4.4 ± 1.8	2–7
Bruising	2 (5.3)	3.5 ± 0.7	3–4
Itching	0 (0)	0	0
Hard lumps	0 (0)	0	0

## 4 Discussion

This study indicates that CaHA acts as an effective and well‐tolerated injectable option for nonsurgical nasal augmentation, with sustained volumetric and aesthetic improvements over a 12‐month follow‐up. The volumetric dynamics observed in this study reveal a distinctive pattern: an initial postinjection increase, a rapid reduction by Month 1, followed by a prolonged plateau phase from Month 1 through Month 9, characterized by overall volume stability with a slight peak around Month 6 that was not statistically significant, and finally a significant decline at Month 12. This pattern provides robust in vivo evidence for CaHA's dual mechanism of action, which combines instantaneous structural filling with progressive tissue regeneration.

The distinctive anatomical features of the Chinese nose, characterized by a relatively lower dorsum and less projected tip compared to Caucasian noses [17, 18], lead to differences in the goals and aesthetic evaluation criteria of nasal shaping between the two populations. Chinese patients typically prioritize a natural, refined appearance, favoring a delicate nasal tip and a straight, well‐defined dorsum [19, 20]. In this research, CaHA fills the middle of the nasal dorsum along the midline to increase the height of the nasal dorsum, and fills the nasal columella and anterior nasal spine to enhance the support of the nasal tip, thereby achieving a coordinated improvement of the midface. Injection within the supraperiosteal plane anchors the CaHA microspheres, reducing filler migration and thereby enhancing the long‐term stability of the correction. From the perspective of patient satisfaction, patient‐reported outcomes at the 12‐month follow‐up showed that over one‐third of subjects remained GAIS responders.

Within the broader landscape of injectable fillers, CaHA is a distinctive option for nasal augmentation. Compared to HA fillers, which offer excellent predictability and reversibility but may lack the necessary G' for strong structural support in the nasal dorsum and are susceptible to enzymatic degradation, CaHA confers higher rigidity and stimulates long‐term collagen‐based support [21]. Our results find that the initial volume change of 2.04 ± 0.67 mL reflects the direct physical space‐occupying effect of the CaHA‐CMC carrier gel [22]. The subsequent volume reduction at 1 month is presumably attributed to partial dissipation of the CMC and post‐procedural edema resolution [23]. From Month 1 to Month 9, volume remained stable, with a modest numerical increase at Month 6 that did not reach statistical significance. This trend is consistent with active neocollagenesis induced by CaHA microspheres, a process supported by established histological findings demonstrating that CaHA microspheres act as a scaffold, releasing calcium ions that recruit macrophages and activate dermal fibroblasts [24, 25, 26]. This cellular interaction promotes sustained synthesis of neocollagen, elastin, and proteoglycans, ultimately contributing to authentic tissue regeneration and volume restoration [22, 25, 27]. CaHA‐induced neocollagenesis compensates for the volume loss resulting from CMC carrier degradation. Between 9 and 12 months, gradual degradation of the CaHA microspheres reduces the stimulus for collagen synthesis, leading to diminished volume increase. These results highlight the biostimulatory efficacy of CaHA, which stands in contrast to the predictable linear degradation characteristic of HA fillers.

In addition, recent studies have shown that HA injection in bony regions could induce bone resorption. Guo et al. reported that a significant reduction in the semimandibular bone thickness in patients who received HA injections for chin augmentation, with the severity of bone loss correlating positively with the injection volume per session [28]. Another case series described a bony erosion in the central symphyseal region directly corresponding to the HA injection site, suggesting a potential pressure‐induced osteolytic process [29]. These findings raise concerns that similar bone resorption could theoretically occur in the nasal region, which also comprises delicate bony structures. In contrast, CaHA exhibits a markedly different biological behavior due to its chemical composition being almost identical to that of human bone mineral. This similarity confers CaHA with an inherent affinity for hard tissues, a property extensively leveraged in craniofacial bone repair and augmentation, by functioning as an effective scaffold and integrating with and reinforcing skeletal structures [30, 31, 32]. This osteogenic potential suggests that CaHA injections at the bone membrane level could potentially counteract or prevent bone resorption, offering a protective effect that HA lacks.

Nasal injections carry a heightened risk of vascular occlusion due to the dense vascular network present in the subcutaneous layer of the nose, including the dorsal nasal artery (DNA), lateral nasal artery (LNA), and columellar artery (CA) (Figure 2B) [33, 34]. Consequently, this study strategically employed a supraperiosteal plane injection technique, which targets a relatively avascular and stable anatomical space [35], thereby significantly mitigating the risk of severe complications such as vascular occlusion. Additionally, the use of blunt‐tip cannulas, as in this study, further enhances safety in this high‐risk zone by reducing the risk of intravascular penetration. In our research, this approach resulted in an excellent safety profile: no vascular complications occurred. Reported adverse reactions, primarily transient, mild‐to‐moderate swelling, and tenderness, are consistent with the expected profile of periosteal injection [11, 36]. The rapid resolution of procedural pain (NRS score of 0 at 60 min) further underscores the tolerability of the CaHA nasal injection. Patients were advised to apply cold compresses to reduce edema and bruising, which resolved spontaneously within around a week. Collectively, the supraperiosteal placement combined with the blunt‐tip cannula procedure established a safety protocol that effectively managed the inherent vascular risks, thereby supporting the procedural viability and patient tolerance of CaHA for nasal augmentation.

In conclusion, this study reveals that CaHA is a safe and effective material for nonsurgical nasal augmentation. Its unique capacity to provide immediate contouring, followed by sustained collagen‐mediated volume restoration, offers a significant benefit for patients seeking durable yet aesthetically natural outcomes in nasal augmentation. However, this study has several limitations. First, it was a single‐center clinical study with a relatively small sample size, which may affect the statistical power and broad applicability of the findings. As a result, although we observed a gradual numerical increase in volume from Month 1 to Month 6 followed by a subsequent decline, the pairwise comparisons among Month 1, Month 3, Month 6, and Month 9 did not reach statistical significance. A larger cohort would be needed to confirm whether these subtle volumetric trends represent consistent population‐level phenomena. Second, the lack of a direct comparator (e.g., HA fillers) precludes definitive conclusions regarding the comparative efficacy and safety of CaHA for nasal augmentation. Third, the exclusive enrollment of female patients, while reflective of the primary demographic seeking this treatment, limits the generalizability of the results to male patients. Finally, the 12‐month follow‐up period, though substantial, may not capture the full duration of effect, as the collagen regeneration induced by CaHA is hypothesized to persist longer.

## 5 Conclusion

CaHA is a safe and effective non‐surgical nasal filler therapy that can achieve significant and durable improvements in nasal volume over a 12‐month follow‐up period. Three‐dimensional volumetric analysis objectively demonstrated its dual mechanism: CaHA provides an immediate postoperative volume enhancement effect, and the gradual formation of new collagen from 1 to 9 months provides continued volume retention. Treatment resulted in high patient satisfaction and a favorable safety profile, with transient and mild‐to‐moderate injection‐site reactions. These findings support CaHA as a valuable regenerative filler option for achieving long‐term, natural‐appearing nasal augmentation.

## Author Contributions

J.W. and W.W. performed the research, analyzed the data, and wrote the manuscript. J.C. and A.Z. contributed to data collection and analysis. P.J. and Y.G. designed the research, supervised the study, and revised the manuscript. All authors read and approved the final manuscript.

## Funding

The authors have nothing to report.

## Disclosure

No AI tools were used for data collection, data analysis, image generation, or figure preparation.

## Consent

Written informed consent was obtained for the use of the two patients' photographs in this article.

## Conflicts of Interest

The authors declare no conflicts of interest.

## Supporting information


**Table S1:** Pairwise comparisons of nasal volume changes over time.
**Figure S1:** Individual patient trajectories of nasal volume changes over the 12‑month follow‑up. Each line represents one of the 24 patients. The graph illustrates the volumetric pattern observed in each subject: an initial decrease from Month 0 to Month 1, followed by a gradual recovery through Month 6, and a subsequent decline through Month 12.

## Data Availability

The data that support the findings of this study are available from the corresponding author upon reasonable request.
