# Supplementary material for: Efficacy and Safety of Calcium Hydroxylapatite for Nasal Augmentation: A 12‐Month Prospective Study in a Chinese Population
Source: J Cosmet Dermatol. 2026 Apr 21;25(4):e70862. doi: 10.1111/jocd.70862 (PMC13100342; doi:10.1111/jocd.70862)

**Supplementary Materials**

**Supplementary Table 1. Pairwise comparisons of nasal volume changes over time**

| Comparison | P value |
| --- | --- |
|  |  |
| 0m vs. 1m | < 0.01 |
| 0m vs. 3m | 0.01 |
| 0m vs. 6m | 0.15 |
| 0m vs. 9m | 0.01 |
| 0m vs. 12m | < 0.01 |
| 1m vs. 3m | 0.48 |
| 1m vs. 6m | 0.08 |
| 1m vs. 9m | 0.59 |
| 1m vs. 12m | 0.75 |
| 3m vs. 6m | 0.94 |
| 3m vs. 9m | > 0.99 |
| 3m vs. 12m | 0.02 |
| 6m vs. 9m | 0.89 |
| 6m vs. 12m | < 0.01 |
| 9m vs. 12m | 0.04 |

**Supplementary Figure 1. Individual patient trajectories of nasal volume changes over the 12‑month follow‑up.** Each line represents one of the 24 patients. The graph illustrates the volumetric pattern observed in each subject: an initial decrease from month 0 to month 1, followed by a gradual recovery through month 6, and a subsequent decline through month 12.


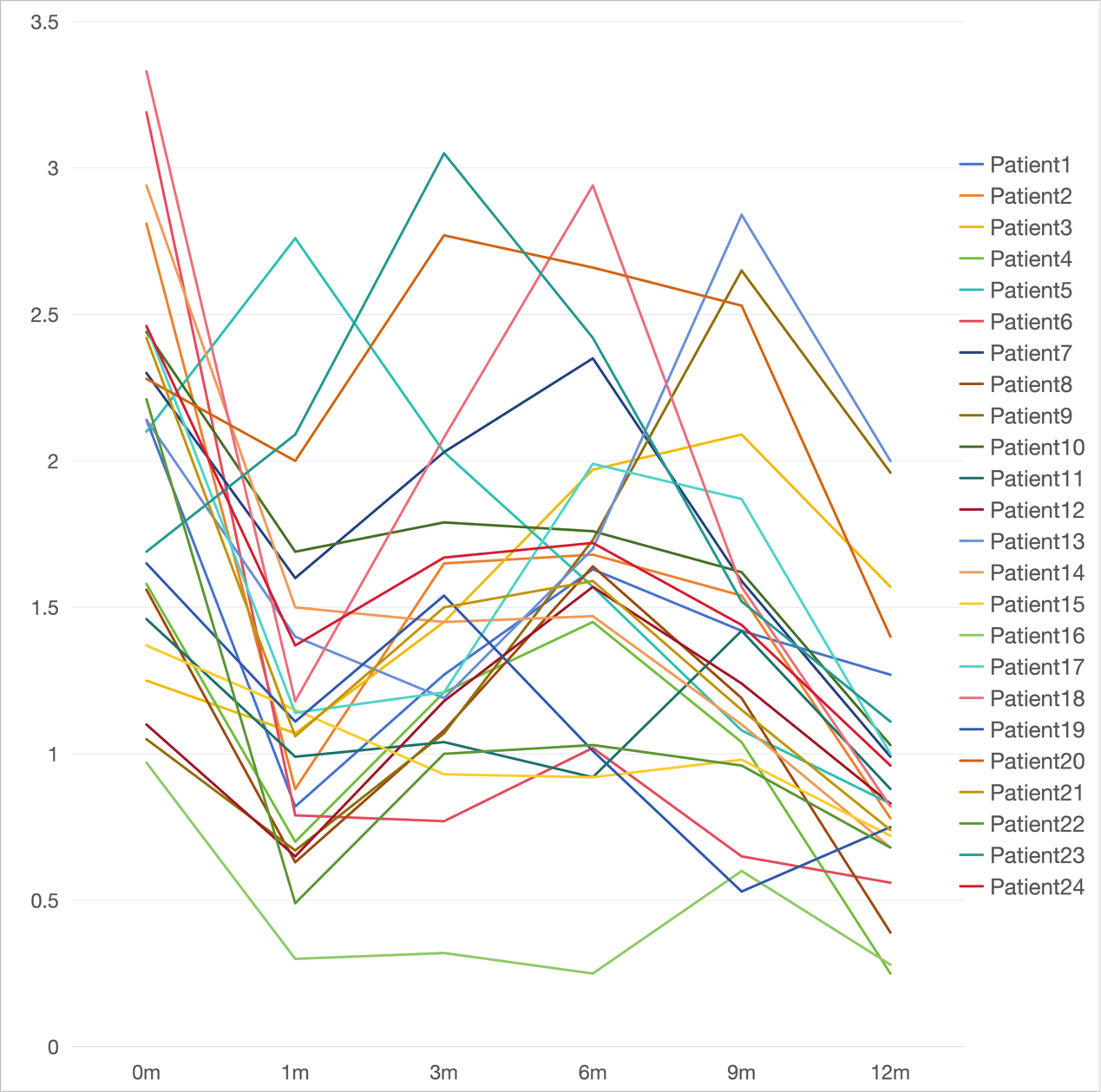

Supplement: Supplementary file 1 — Table S1: Pairwise comparisons of nasal volume changes over time. Figure S1: Individual patient trajectories of nasal volume changes over the 12‑month follow‑up. Each line represents one of the 24 patients. The graph illustrates the volumetric pattern observed in each subject: an initial decrease from Month 0 to Month 1, followed by a gradual recovery through Month 6, and a subsequent decline through Month 12. [file JOCD-25-e70862-s001.docx]
